# Prevalence of Class 1 Integrons and Extended Spectrum Beta Lactamases among Multi-Drug Resistant *Escherichia coli* Isolates from North of Iran

**DOI:** 10.7508/ibj.2015.04.007

**Published:** 2015-10

**Authors:** Mohammad Javad Mehdipour Moghaddam, Adeleh Alsadat Mirbagheri, Zivar Salehi, Seyyed Mahmood Habibzade

**Affiliations:** 1 *Dept. of Biology, Faculty of Science, University of Guilan, Rasht, Iran; *; 2 *University of Guilan, University Campus 2, Rasht, Iran; *; 3 *Razi Pathobiology Laboratory, Rasht, Iran*

**Keywords:** Antibiotic, Integrons, *Escherichia coli*

## Abstract

**Background:**

Extended spectrum beta lactamases (ESBLs) are an important cause of transferable multidrug resistance (MDR) in gram-negative bacteria. The most described ESBL genes are generally found within integron-like structures as mobile genetic elements. The aim of this study was to identify the accompanying of class 1 integrons and ESBLs in the MDR *E. coli* isolates.

**Methods:**

Susceptibility to antimicrobial agents was determined for 33 *E. coli *strains by the disk diffusion method. Double-disk synergy test was applied for screening ESBL. To identify the strains carrying integrons, the conserved regions of integron-encoded integrase gene *intI1 *were amplified. For detection of gene cassettes, 5′CS and 3′CS primers were used.

**Results:**

All *E. coli* isolates were identified as multi-drug resistant. More than 50% of the isolates were resistant to tetracycline, cephalothin, cefuroxime, amoxicillin-clavulanic acid, and third generation cephalosporines. Nearly all of the isolates displayed sensitivity to piperacillin. There was a significant correlation between production of ESBL and resistance to all antibiotics except for ciprofloxacin and piperacillin (*P *< 0.01). Thirty two MDR strains (97%) included class 1 integron, and some isolates that included integrons were similar in the size of gene cassettes. The isolates were different in the resistance profiles; however, some others had similar resistance profiles. Of eight ESBL positive isolates, seven (87.5%) carried class 1 integrons.

**Conclusion:**

Class 1 integrons were frequent in MDR and also ESBL-producing *E. coli *isolates. High prevalence of class 1 integrons confirms that integron-mediated antimicrobial gene cassettes are important in *E. coli* resistance profile.

## INTRODUCTION

Despite accessibility to different antibiotics, Urinary tract infections (UTIs) are the second most frequent community-acquired adult infection and the main cause of nosocomial infection [1]. *E. coli *is one of the major causes of nosocomial infections, especially in patients in intensive care units and the main cause of UTI in developed world, accounting for 70-90% of uncomplicated UTIs [2].

Understanding the molecular mechanism of resistance genes may help to the introduction of new antimicrobial strategies and some preventive procedures to prevent further spreading of resistance determinants among the pathogens [3]. Several resistance genes encode different mechanisms of drug resistance in bacterial genomes and in extra-chromosomal pieces of DNA [4].

Multidrug resistance (MDR) is a major problem worldwide and encoded by resistance genes exists on integrons. Integrons are mobile genetic elements considered to be responsible for the MDR transfer. Most of the integrons reported in clinical isolates are class 1 inegrons. Class 1 integrons carry integrase gene (*intI1*), which codes for the site-specific recombinase responsible for cassette insertion [5, 6]. Integrase gene also includes  the *attI1* site, where the cassettes are integrated and a promoter, Pc, is  responsible for the transcription of the cassette-encoded genes [5, 7]. Gene cassette contains a single antibiotic resistance gene and a 59-base element (or *attC* site) downstream of the gene, which is responsible for recombination events [8]. 

Extended spectrum beta lactamases (ESBLs) are a group of enzymes that are common among Enterobacteriaceae [9]. They are the increasingly important cause of transferable MDR in Gram-negative bacteria throughout the world. ESBLs also have the ability to hydrolyze third and fourth generation cephalosporins and monobactams. ESBL-producing strains are prevented by lactamase inhibitors (clavulanic acid, sulbactam, and tazobactam) [10].

ESBL-encoding genes are generally located on conjugative plasmids (such as *blaTEM *or *blaSHV*), although many of the most newly described ESBL genes are usually found within integron-like structures (such as *blaCTX*-*M*, *blaGES*, or *blaVEB*-*1*) [11-13]. On the other hand, ESBL-producing isolates usually show resistance to other antibiotics including aminoglyc-sides, tetracyclines, chloramphenicol, trimethoprim, sulfonamides, or quinolones. Resistance to these antibiotics is mostly related to presence of different resistance genes on plasmids, transposons, or integrons as transferable elements or genetic structures generated by combinatorial evolution of different interactive pieces [13-15]. The presence of ESBL genes on integrons can facilitate the distribution of such genetic elements [11, 12].

The aim of this study was to identify whether there are mechanisms of resistance in the *E. coli* isolates causing UTIs from north of Iran or not.

## MATERIALS AND METHODS

***Sample collection and identification of bacterial strains. ***Urine samples were collected from appropriate patients in early morning mid-stream using sterile, wide mouthed glass bottles with screw cap tops between May and July 2012. Samples were maintained in an icebox until laboratory analysis. It did not last more than one hour between sample collection and sample analysis. Urine samples were cultured on nutrient, blood and MacConkey agar plates and incubated at 37°C for 18-24 h. The usual bacteriological methods were applied for cultivation, isolation and identification of isolates from urine samples. The isolates were stored at -70ºC in a tryptic soy broth containing 15% glycerol until processing. The isolates were entitled as E1 to E33. 

***Antimicrobial susceptibility test. ***According to *Clinical and Laboratory Standards Institute (*CLSI) [16], disc diffusion test was applied to identify the susceptibility of the isolates to the following antimicrobials: piperacillin (100 μg), streptomycin (10 μg), tetracycline (30 μg), chloramphenicol (30 μg), cefepime (30 μg), ceftriaxone (30 μg), ceftazidime (30 μg), cephalothin (30 μg), cefotaxime (30 μg), cefuroxime (30 μg), imipenem (10 μg), amoxicillin-clavulanic acid (20/10 μg), and ciprofloxacin (5 μg). *E. coli* ATCC 25922 and ATCC 35218 were used as the reference strains to control the quality of the applied antimicrobial agents. MDRs were described as resistance to three or more antimicrobials. 

***Phenotypic detection of ESBLs*** **. **Double-disk synergy tests were performed by placing disks of ceftazidime, cefotaxime, and cefepime (30 μg each) at 30 or 20 mm distance (center to center) from a disk containing amoxicillin (20 μg) and clavulanic acid (10 μg). When the cephalosporin zone was expanded by the clavulanate, ESBL production was supposed. It means that the zones produced by the disks with clavulanate were ≥5 mm larger than those without any inhibitor [16].

***Detection of class 1 integrons by PCR. ***The existence of class 1 integrons was identified by PCR using specific primers for the integron integrase genes *intI1* (Table 1). A single colony of each isolate was suspended in 25 ml reaction mixture containing 2.5 ml 10× PCR, 1.5 ml 50 mM MgCl_2_, 2 ml 2.5 mM dNTP, 1 ml primer (forward and reverse) together with 1 unit *Taq *DNA polymerase (5 U/ml). Volume of the reaction mixture was adjusted by adding filtered deionized water. PCR assays were performed in a DNA thermal cycler (BioRad, USA). The PCR conditions were initial denaturation at 94ºC for 12 min, followed by 30 cycles of 1 min at 94ºC, 30 s at 60ºC for annealing, 2 min at 72ºC for elongation, and final extension was conducted at 72°C for 10 min. A reagent blank was included in every PCR assay containing all components of the reaction mixture except for the bacteria. ATCC *E. coli *25922 strain was used as negative control for all PCR assays, and *E. coli *ur-31 was used as positive control for *intI1* gene. PCR products were subjected to horizontal gel electrophoresis on 1% agarose gel (type II, Sigma, USA) in Tris-borate EDTA buffer at 100 volt (50 mA) at room temperature for 1 h. DNA bands were visualized by gel staining with ethidiumbromide (0.5 mg/ml) for 30 min and then photographed [17].

**Table 1:** Oligonucleotide primers used in the PCR analysis

**Primer**	**Oligoneucleotide sequence (5** **´** **-3** **´** **)**	**Amplicon size (bp)**
Int1F	GGTCAAGGATCTGGATTTCG	491
Int1R	ACATGCGTGTAAATCATCGTC	491
5́-CS	GGCATCCAAGCAGCAAG	variable
3́-CS	AAGCAGACTTGACCTGA	variable

**Table 2:** Antibiotic resistance profiles for ESBL and non-ESBL* E. coli* isolates

**Antimicrobial agents (µg)**	**Diffusion zone (mm)**	**ESBL isolates** **(n = 8)**				**Non-ESBL isolates** **(n = 25)**				***P *** **value**
		S* (%)	I* (%)	R* (%)		**S (%)**	**I (%)**	**R (%)**		
Streptomycin (10 )	≤11	0 (0)	5 (62.5)	3 (37.5)		4 (16)	12 (48)	9 (36)		0.01
Cefepime (30)	≤14	1 (12.5)	2 (25)	5 (62.5)		13 (52)	3 (12)	9 (36)		0.01
Ceftriaxone (30)	≤13	1 (12.5)	0 (0)	7 (87.5)		9 (36)	4 (16)	12 (48)		0.01
Amoxicillin-clavulanic acid (20/10)	≤13	0 (0)	0 (0)	8 (100)		1 (4)	3 (12)	21 (84)		0.01
Chloramphenicol (30)	≤12	3 (37.5)	0 (0)	5 (62.5)		14 (56)	1 (4)	10 (40)		0.01
Ceftazidime (30)	≤14	2 (25)	2 (25)	4 (50)		10 (40)	2 (8)	13 (52)		0.01
Ciprofloxacin (5)	≤15	3 (37.5)	2 (25)	3 (37.5)		12 (48)	5 (20)	8 (32)		Ns*
Imipenem (10)	≤15	2 (25)	1 (12.5)	5 (62.5)		16 (64)	3 (12)	6 (24)		0.01
Cephalothin (30)	≤14	0 (0)	0 (0)	8 (100)		5 (20)	0 (0)	20 (80)		0.01
Cefotaxime (30)	≤14	1 (12.5)	0 (0)	7 (87.5)		7 (28)	5 (20)	13 (52)		0.01
Tetracyclines (10)	≤11	0 (0)	0 (0)	8 (100)		4 (16)	2 (8)	19 (76)		0.01
Cefuroxime (30)	≤14	0 (0)	1 (12.5)	7 (87.5)		6 (24)	3 (12)	16 (64)		0.01
Piperacillin(100)	≤11	7 (87.5)	1 (12.5)	0 (0)		22 (88)	2 (8)	1 (4)		Ns

***Detection of gene cassettes. ***To characterize the gene cassettes inserted in the class 1 integrons, the fragments including the cassette regions were amplified by PCR. The primers used to amplify cassettes of class 1 integrons were 5́CS and 3́CS (Table 1) as mentioned previously [18].

## RESULTS

***Antimicrobial resistance. ***The resistance patterns of the 33 urinary isolates are shown in the Table 2. All *E. coli* isolates (33 strains) were identified as multi-drug resistant. More than 50% of the isolates were resistant to tetracycline, cephalothin, cefuroxime, amoxicillin-clavulanic acid, and third generation cephalosporines. Nearly all isolates displayed sensitivity to piperacillin.

***ESBL screening. ***Screening of ESBLs by double-disc synergy test indicated that eight isolates (24%) were ESBL producers. Phenotypic confirmatory disc diffusion test confirmed that 8 of the 33 isolates were ESBL producers. All ESBL isolates were completely resistant to tetracyclines, cephalothin and amoxicillin-clavulanic acid, but rates of resistance to other antibiotics were as bellow: amoxicillin-clavulanic acid 100%, cephalothin 100%, tetracycline 100%, ceftriaxone 87.5%, cefotaxime 87.5%, cefuroxime 87.5%, chloramphenicol 62.5%, cefepime 62.5%, imipenem 62%, ceftazidime 50%, streptomycin 37.5%, ciprofloxacin 37.5%. Antibiotic resistance profiles for ESBL and non-ESBL isolates are shown in Table 2. According to the Table, there was a significant correlation between ESBL production and resistance to all antibiotics except for ciprofloxacin and piperacillin (*P* < 0.01). 

**Fig.1:**
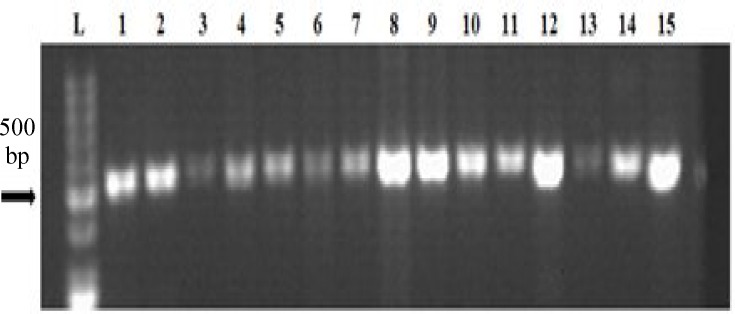
PCR amplification of *IntI1* gene among some MDR *E. coli* isolates on 0.7% agarose gel. In all isolates, a fragment of 483 bp was detected. Lane L: DNA ladder. Lane 1, *E. coli* ATCC 25922 as a positive control for *IntI1 *gene; Lanes 2-15, Amplified gene of *IntI1 *in the tested clinical isolates

**Fig. 2:**
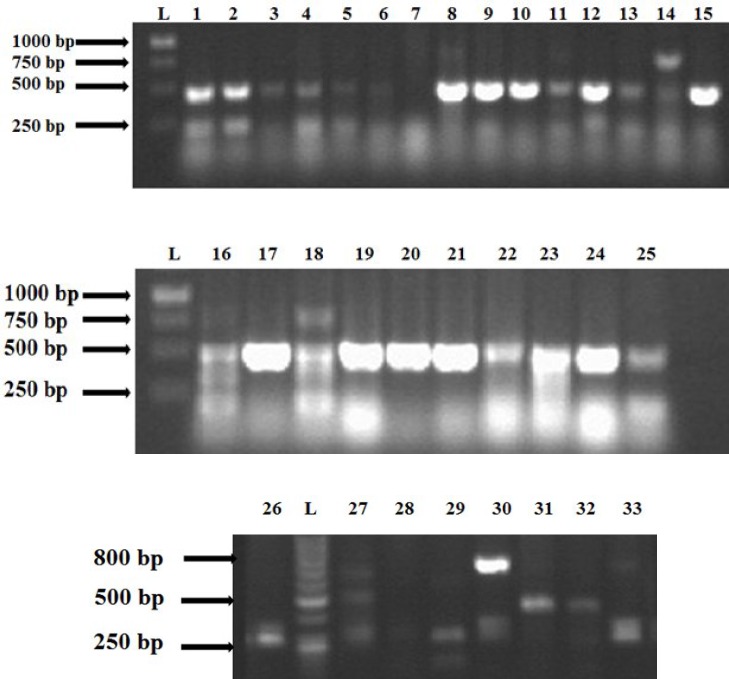
The amplified gene cassettes with different sizes in the tested clinical isolates (1-33). L, DNA ladder

***Detection of class 1 integrons and gene cassettes. ***PCR analysis revealed that 32 strains (97%) contained *IntI1* gene (Fig. 1). Among the eight ESBL-producing strains, seven had class 1 integrons. The detected fragment size was 483 bp. The strains including integrons had variable gene cassettes (Fig. 2). One or maximum four gene cassettes was carried by class 1 integrons. Based on PCR amplification with primers 5́CS and 3́CS, the inserted gene cassettes of class 1 integrons were identified as 100, 250, 500, and 700 bp. The isolates similar in the number of gene cassettes were compared based on the results of disk diffusion (Table 3). Some isolates were similar in the size of gene cassettes, which seemed that they were different in the resistance profiles; however, some other had similar resistance profiles.

## DISSCUSION

Multi-drug resistant bacteria have been recently a problem in hospitalized patients worldwide. The frequency of MDR among clinical isolates is highly variable throughout the world and in different geographic areas and is rapidly changing according to time [19]. MDRs encoded by linked resistance genes exist  on integrons, which are potentially movable genetic elements supposed to be involved in transferring MDR. This study was designed to examine the drug resistance pattern, the frequency of the class 1 integrons and ESBLs among the MDR *E. coli *isolates in UTI patients in north of Iran, Rasht. 

Based on the results, *E*. *coli *was the most frequent microorganism isolated from urine samples. Other investigators also reported that *E*. *coli *was the most commonly isolated aerobic microorganism from UTIs [20, 21]. In our study, similar to Salem *et al.* survey [17], all *E. coli* isolates were recognized as MDR. In Ahangarzadeh Rezaee *et al.* study [22], 84.2% of the isolates were multi-drug resistant.

Nearly all *E. coli *isolates in this survey were sensitive to p*iperacillin, while* some of them were resistant to other antimicrobials. The isolates displayed variable patterns of resistance to different antibiotics. More than 50% of the isolates were resistant to tetracycline, cephalothin, cefuroxime, amoxicillin-clavulanic acid, and third generation cephalosporines. According to Salem *et al. *[17], all *E. coli *isolates displayed resistance to penicillins, amoxicillin, cephalexin, and chloramphenicol. The isolates showed variable patterns of resistance to tetracycline, sulfamethoxazole-trimethoprim, erythromycin, and quinolone group.

In Ahangarzadeh Rezaee *et al*. [22] study on *E*. *coli *isolates from north-west of Iran, antibiotic resistance patterns were as follows: amoxicillin 99.3%, cephalothin 77.8%, co-trimoxazole 75%, tetracycline 72.8%, nalidixic acid 60.7%, norfloxacin 50.7%, ciprofloxacin 47.6%, ceftazidime 46.4%, gentamicin 33.6%, chloramphenicol 20.7%, nitrofurantoin 12.9%, amikacin 12.1%, and imipenem 1.4%.

The antimicrobial resistance genes located on integron-like structures are being dramatically reported worldwide [11]. Most of the newly described ESBL genes are generally found within integron-like structures (such as *blaCTX*-*M*, *blaGES*, or *blaVEB*-*1*) [11, 13]. On the other hand, ESBL-producing isolates are usually resistant to other antibiotics, such as aminoglycosides, tetracyclines, chloramphenicol etc. The fact that ESBL genes could be acquired by strains including particular integrons enlarges the possibilities of the selection of these strains by a variety of different antimicrobials. In addition, ESBL genes can be located on integrons, which may simplify the distribution of such genetic elements [11, 12].

**Table 3:** The relation between resistance profiles and the number of gene cassettes

**Isolates**	**Resistance**	**Number of** **gene cassettes**	**Band(s)** **size (bp)**
E6, E7	Amoxicillin-clavulanic acid Tetracyclines	1	250
			
E1, E2	Chloramphenicol Tetracyclines	2	250, 500
			
E17, E19 E22, E23, E24, E25	Streptomycin Amoxicillin-clavulanic acid Cephalothin Tetracyclines Ceftriaxone Chloramphenicol Cephalothin Tetracyclines	2	100, 500
			
E8, E9	Amoxicillin-clavulanic acid Ceftazidime Cephalothin Tetracyclines Cefuroxime	2	250, 500
			
E12, E15	Amoxicillin-clavulanic acid Ceftazidime Cephalothin Cefotaxime	2	250, 500
			
E16, E18	Amoxicillin-clavulanic acid Ceftriaxone Ceftazidime Cephalothin Cefotaxime Cefuroxime	4	100, 250,500,700

For detection of class 1 integrons and ESBLs in this study, PCR amplification of *intI1* gene and Double-disk synergy test method were used, respectively. Based on the results, 97% and 24% of the isolates contained class 1 integrons (483 bp in size) and ESBLs, respectively, which is indicative of very high frequency of occurrence of class 1 integrons in *E. coli *strains. Also, nearly all ESBL isolates carried class 1 integrons. 

In 2008, Phongpaichit *et al*. [23] studied the susceptibility of 175 *E. coli* isolates from stools against 12 antimicrobial agents and also the presence of class 1 integrons. Their results indicated that 63% of the isolates included class 1 integrons, and the majority of the isolates (85%) were resistant to at least one antimicrobial agent with the following resistance rate: streptomycin 66%, tetracycline 60%, sulfamethoxazole 59%, ampicillin 52%, trimethoprim-sulfamethoxazole 47%, kanamycin 30%, nalidixic acid 27%, ciprofloxacin 23%, norfloxacin 22%, amoxicillin-clavulanic acid 16%, gentamicin 8%, and amikacin 2%*.*

The presence of class 1 and class 2 integrons were also investigated in Salem *et al*. study [17]. The results indicated that the class 1 integrons were observed in *E. coli *isolates (54%), and *IntI1* gene yielded a DNA fragment of 1900 bp upon amplification by PCR, but class 2 integrons showed negative results. The absence of *IntI2* gene may be attributed that class 2 integrons found in 4 to 20% uropathogenic *E. coli *strains [24, 25] as well as in other human pathogens [26], other animal pathogens [27], and various commensal bacteria [28, 29]. Essen-Zandbergen *et al*. [30], reported that among all *E. coli* isolated from animals, class 1 integrons were found in 76%, and the size of gene cassettes were 600, 1000, 1550, 2000, 2200, and 2500 bp. Martinez-Freijo *et al*. [31] study represented that 62% of *E. coli* isolates were integron 1-positive and had different sizes of inserted gene cassette, including 1500, 1600, 1800, 2000, and 3000 bp. In Farshad *et al. *[32] study performed in Shiraz (Iran), of 96 *E. coli* isolates from urine samples tested, 6.25% of the strains were the integron 1-positive isolates. In other words, the prevalence of class 1 integrons among the isolates was low.

In Madrid (Spain), Machado *et al*. [33] investigated the existence of class 1 integrons in ESBL-producing in 133 *E. coli* isolates and reported that 39% of the isolates were ESBL producing, and class 1 integrons were more common among 67% of the isolates. Thus, based on their study, a significant relationship was not observed between the spread of genetic elements encoding ESBL and the dispersion of integrons in these isolates.

Karimi *et al.* [34] detected class 1 integrons in ESBL producing in 100 *E. coli* isolates in Tehran, Iran. Thirty *E. coli *isolates were positive for ESBLs, and the frequency of class 1 integrons was 52%. Also, 54% of ESBL producers had class 1 integrons. The detected fragment of *intI1* was 9000 bp. 

In a survey by Al-Assil *et al*. [35] in Aleppo (Syria), 61.33% of uropathogenic *E. coli *were ESBL producers, and class 1 integrons were detected in 54.6% of them. Prevalence of class 1 integrons in ESBL isolates was 34%. 

In conclusion, this study demonstrates that unlike other researches, the prevalence of class 1 integrons in ESBL producing *E. coli* strains is very common. The results of the current study also indicated that there are at least two resistance mechanisms in our isolates, and they can be transferred to other clinical strains, then it is very important to identify and control the resistant strains. Increased resistance may be related to the lack of proper research, abuse of chemotherapeutic agents, public misuse of antibiotics, and little or no preventive measure. Thus, piperacillin can be used as a therapeutic agent or the most effective antibiotic in controlling UTI infections in the patients infected by ESB-producing *E. coli* carrying class 1 integrons in Rasht (Guilan Province, Iran). Nevertheless, more studies are needed to be performed in this area.

## References

[1] Huang ES, Stafford RS (2002). National patterns in the treatment of urinary tract infections in women by ambulatory care physicians. Arch Intern Med.

[2] Gupta K, Sahm DF, Mayfield D (2001). Antimicrobial resistance among uropathogens that cause community-acquired urinary tract infections in women: a nationwide analysis. Clin Infect Dis.

[3] Shapiro JA (1999). Views about evolution are evolving. Am Soc Microbiol News.

[4] Soulsby L (2005). Resistance to antimicrobials in humans and animals. Br J Med.

[5] Roy Chowdhury P, Ingold A, Vanegas N, Martínez E, Merlino J, Merkier AK (2011). Dissemination of multiple drug resistance genes by class 1 integrons in Klebsiella pneumoniae isolates from four countries: a comparative study. Antimicrob Agents Chemother.

[6] Betteridge T, Partridge SR, Iredell JR, Stokes HW (2011). Genetic context and structural diversity of class 1 integrons from human commensal bacteria in a hospital intensive care unit. Antimicrob Agents Chemother.

[7] Stalder T, Barraud O, Casellas M, Dagot C, Ploy M (2012). Integron involvement in environmental spread of antibiotic resistance. Front Microbiol.

[8] Collis CM, Kim MJ, Stokes HW, Hall RM (2002). Integron-encoded IntI integrases preferentially recognize the adjacent cognate attI site in recombination with a 59-be site. Mol Microbiol.

[9] Poole K (2004). Resistance to β-Lactam antibiotics. Cell Mol Life Sci.

[10] Pitout JDD, Hamilton N, Church DL, Nordmann P, Poirel L (2007). Development and clinical validation of a molecular diagnostic assay to detect CTX-M-type β-lactamases in Enterobacteriaceae. Clin Microbiol Infect.

[11] Bonnet R (2004). Growing group of extended-spectrum beta-lactamases: the CTX-M enzymes. Antimicrob Agents Chemother.

[12] Canto´n R, Coque TM, Baquero F (2003). Multi-resistant gram-negative bacilli: from epidemics to endemics. Curr Opin Infect Dis.

[13] Jacoby GA, Sutton L (1991). Properties of plasmids responsible for production of extended-spectrum β-lactamases. Antimicrob Agents Chemother.

[14] Preston KE, Graffunder EM, Evans AM, Venezia RA (2003). Survey of plasmid-associated genetic markers in Enterobacteriaceae with reduced susceptibilities to cephalosporins. Antimicrob Agents Chemother.

[15] Villa L, Pezzella C, Tosini F, Visca P, Petrucca A, Carattoli A (2000). Multiple-antibiotic resistance mediated by structurally related IncL/M plasmids carrying an extended-spectrum beta-lactamase gene and a class 1 integron. Antimicrob Agents Chemother.

[16] Clinical and Laboratory Standards Institute (CLSI) Performance standards for antimicrobial susceptibility testing: Twenty-first information supplement M100-S21.

[17] M. Salem M, Muharram M, Alhosiny IM (2010). Distribution of classes 1 and 2 integrons among multi drug resistant E. coli isolated from hospitalized patients with urinary tract infection in Cairo, Egypt. Aus J Basic Appl Sci.

[18] Le´ vesque C, Piche´ L, Larose C, Roy PH (1995). PCR mapping of integrons reveals several novel combinations of resistance genes. Antimicrob Agents Chemother.

[19] Magiorakos AP, Srinivasan A, Carey RB, Carmeli Y, Falagas ME (2012). Multidrug-resistant, extensively drug-resistant and pandrugresistant bacteria: an international expert proposal for interim standard definitions for acquired resistance. Clin Microbiol Infect.

[20] Al-Agamy MH, Ashour MS, Wiegand I (2006). First description of CTX-M β-lactamase-producing clinical Escherichia coli isolates from Egypt. Int J Ant Microb Agents.

[21] Asour HM, Elsharif A (2009). Species distribution and antimicrobial susceptibility of Gram-negative aerobic bacteria in hospitalized cancer patients. J Trans Med.

[22] Ahangarzadeh Rezaee M, Sheikhalizadeh V, Hasani A (2011). Detection of Integrons among multi-drug resistant (MDR) Escherichia Coli strains isolated from clinical specimens in northern wset of Iran. Braz J Microbiol.

[23] Phongpaichit S, Wuttananupan K, Samasanti W (2008). Class 1 integrons and multidrug resistance among Escherichia coli isolates from human stools. Southeast Asian J Trop M.

[24] Yu HS, Lee JC, Kang HY, Ro DW, Chung JY, Jeong YS (2003). Changes in gene cassettes of class 1 integrons among Escherichia coli isolates from urine specimens collected in Korea during the last two decades. J Clin Microbiol.

[25] Solberg OD, Ajiboye RM, Riley LW (2006). Origin of class 1 and 2 integrons and gene cassettes in a population-based sample of uropathogenic Escherichia coli. J Clin Microbiol.

[26] Ranjbar R, Aleo A, Giammanco GM, Dionisi AM, Sadeghifard N, Mammina C (2007). Genetic relatedness among isolates of Shigella sonnei carrying class 2 integrons in Tehran, Iran, 2002–2003. BMC Infect Dis.

[27] Kadlec K, Schwarz S (2008). Analysis and distribution of class 1 and class 2 integrons and associated gene cassettes among Escherichia coli isolates from swine, horses, cats and dogs collected in the BfT-GermVet monitoring study. J Antimicrob Chemother.

[28] Barlow RS, Pemberton JM, Desmarchelier PM, Gobius KS (2004). Isolation and characterization of integron-containing bacteria without antibiotic selection. Antimicrob Agents Chemother.

[29] Barlow RS, Fegan N, Gobius KS (2008). A comparison of antibiotic resistance integrons in cattle from separate beef meat production systems at slaughter. J Appl Microbiol.

[30] Essen-Zandbergen AV, Smith H, Veldman K, Mevius D (2007). Occurrence and characteristics of class 1, 2 and 3 integrons in Escherichia coli, Salmonella and Campylobacter spp. in the Netherlands. J Antimicrob Chemother.

[31] Martinez-Freijo P, Fluit AC, J Schmitz F, Grek VSC, Verhoef J, Jones ME (1998). Class 1 integrons in gram-negative isolates from different European hospitals and association with decreased susceptibility to multiple antibiotic compounds. J Antimicrob Chemother.

[32] Frashad S, Japoni A, Hosseini M (2008). Low distribution of integrons among multidrug resistance E. coli strains isolated from children with community-acquired urinary tract infections in Shiraz, Iran. Pol J Microbiol.

[33] Machado E, Canto´n R, Baquero F, Gala´n GC, Rolla´n A, Peixe L (2005). Integron content of extended-spectrum-β-Lactamase-producing Escherichia coli strains over 12 years in a single hospital in madrid, Spain. J Agents Chemother.

[34] Karimi A, Rahbar M, Fallah F, Navidinia M, Malekan MA (2012). Detection of integron elements and gene groups encoding ESBLs and their prevalence in Escherichia coli and Klebsiella isolated from urine samples by PCR method. Afr J Microbiol Res.

[35] Al-Assil B, Mahfoud M, Hamzeh, AR (2013). First report on class 1 integrons and trimethoprim-resistance genes from dfrA group in uropathogenic E. coli (UPEC) from the aleppo area in Syria. Mob Gen Elements.

